# Mental health status and its associated factors among women with fertility issues visiting fertility center in Kathmandu Valley: A cross-sectional study

**DOI:** 10.1371/journal.pgph.0005487

**Published:** 2026-08-27

**Authors:** Pragya Kunwar, Amrita Ghimire, Aakriti Bharati, Amrit Bist, Laxmi Gautam

**Affiliations:** 1 School of Public Health, B.P. Koirala Institute of Health Sciences, Dharan, Nepal; 2 Department of Public Health, Manmohan Memorial Institute of Health Sciences, Kathmandu, Nepal; 3 Vatsalya Health Care Pvt. Ltd., Kathmandu, Nepal; 4 School of Public Health Sciences, Patan Academy of Health Sciences, Lalitpur, Nepal; University of Balamand Faculty of Health Sciences, LEBANON

## Abstract

Infertility poses a multidimensional burden, especially for women in low- and middle-income countries, where it is often associated with social stigma, emotional distress, and limited support. This study aimed to assess the mental health status and its associated factors among women with fertility issues visiting fertility center in the Kathmandu Valley. A cross-sectional study was conducted from October 2023 to April 2024, and participant data collected from November 2023 to February 2024, among 143 women of reproductive age (18–49 years). Nepali versions of the DASS-21 were administered to to collect data. Descriptive and inferential statistics were performed using SPSS version 22.0. The logistic regression model was applied to examine the factors associated with the outcome variable using an adjusted odds ratio with a 95% CI, and a p-value < 0.05 was considered statistically significant. More than four-fifths of respondents (82.5%) reported experiencing some level of anxiety, followed by 51.7% with stress and 35.0% with depression. Women who felt insecure about not having a child had significantly higher odds of stress (AOR = 3.19), while those facing social stigmas also had elevated odds of stress (AOR = 2.91) and anxiety (AOR = 1.55). Experiencing abuse was strongly associated with stress (AOR = 12.76). Women who had never conceived were more likely to experience anxiety (AOR = 2.56), as were those facing financial burdens (AOR = 4.78). Depression was more likely among women dissatisfied with their married life (AOR = 4.30) and among those with a female factor of infertility (AOR = 14.14) compared to unexplained infertility. Increased odds of depression were also associated with longer treatment duration (>5 years) (AOR = 3.34) and higher treatment cost (>5 lakhs) (AOR = 2.71). Anxiety, stress and depression were found to be common among women with fertility issues. Higher psychological distress requires prompt actions to address the mental health needs of women who are battling with infertility.

## Background

Infertility has been recognized as a public health issue worldwide. According to the World Health Organization (WHO), infertility is defined as a disease of the male or female reproductive system defined by the failure to achieve a pregnancy after 12 months or more of regular unprotected sexual intercourse [1,2]. It is estimated that approximately one in every six people of reproductive age worldwide may experience infertility during their lifetime [1]. It is believed that a significant proportion of couples, approximately 21% experience phases of infertility throughout their lives [3]. According to the World Health Organization and recent global burden studies, infertility affects an estimated 15–20% of couples in LMICs, with some studies reporting rates up to 30% in sub-Saharan Africa [1]. It is a multidimensional stressor with several kinds of emotional adjustments [4]. It is associated with different problems like dysfunction in sexual relationships, anxiety, depression and difficulties in marital life [5].

Globally, depression affects approximately 4–6% of general population, with WHO estimating about 5% of adults (280 million people) living with depression worldwide [6]. The WHO’s depression statistics over the world vary by sex, with the prevalence among females being 50% higher than males [6–8]. Depression is one of the most common psychological consequences of infertility, and its severity appears to be shaped, in part, by the local social and cultural context [6,7,9]. In countries such as the United States, United Kingdom, and Australia, the occurrence rate of depression ranges from 5-12% for men and 10–25% for women who are facing infertility [10]. Notably, major depression appears to be two to three times more prevalent in women compared to men during infertility [11]. The prevalence of depression among women with fertility issues as found in studies by Lok et al., Fatemeh et al., and Al-Homaidan was 33%, 40.8%, and 53.8%, respectively [12–14]. Depression and anxiety are the most commonly reported mental health issues among people living with infertility. A survey by Ramezanzadeh et al. (2004) found that after 4–6 years of infertility duration, depression and anxiety are most prevalent mental issues among infertile population with severe depression reported in individuals who had been infertile for 7–9 years; among whom 40.8% of women with fertility issues had depression, and 86.8% had anxiety [13]. A study among women with fertility issues in the Tertiary Care Hospital of Karachi, Pakistan, indicated that infertile (primary infertile group) women had the highest percentage of depression (severe-58.2%), anxiety (severe-57.3%), and stress (severe- 50.0%) [15]. A similar conclusion was reached in Bangladesh (54.5%) and Iran studies (58.0%), and a lower percentage was identified in studies in Sweden (14.8%), Saudi Arabia (21.8%), China (45.0%), and Nepal (40.0%) [16–19]. Studies also shows that individuals undergoing fertility treatment often experience significant psychological distress—including anxiety and depression related to repeated treatment failure, mood disturbances linked to hormonal medications, financial strain from prolonged care, and uncertainty during diagnosis and treatment highlighting the need for integrated psychosocial support within infertility services [17,19].

In addition, infertility can have negative social and psychological implications for an individual, ranging from divorce to more subtle types of social stigma that lead to isolation and mental distress [9]. A study in Pakistan showed that more than 67.7% of participants had disputes in interpersonal relationships [20]. One of the biggest challenges that women with fertility issues face is dealing with feelings of jealousy and envy when learning of other women’s pregnancies or being in the presence of others who have infants [3]. Some women might hide their pain from healthcare experts because they are anxious, nervous of being criticized, or afraid of being labeled as insane [9]. According to reports, up to 13% of women develop passive thoughts of suicide following an unsuccessful In-Vitro Fertilization (IVF) effort [9].

Moreover, the social repercussions for South Asian women, such as women from Nepal, living in patriarchal cultures, where motherhood is often the sole way for women to advance in the family and community, are potentially far greater than for women in Western communities [21]. The woman may be held responsible for infertility regardless of whether the medical cause is with her or with her spouse [22]. The marriage relationship is frequently compromised, even leading to intimate partner violence and distress [22]. In reality, women with fertility issues face the constant threat of divorce or remaining in a polygamous family, which forces them to lower their quality of life and endure significant socioeconomic and psychological hardships [23]. Despite this, there has been no successful program created in Nepal to address the many reasons for infertility [24]. Infertility has received little attention in Nepal because it is not a life-threatening condition, although it causes severe community health issues such as depression, anxiety, spousal violence, and social isolation [24].

Mental health status in the context of this study refers to the presence and severity of clinically relevant symptoms of depression, anxiety, and stress, as assessed using standardized psychometric instruments. This cross-sectional study assessed mental health status and its associated factors among women with fertility issues visiting fertility center in Kathmandu Valley. This research can aid in the development of specific psychological interventions, which are currently lacking in the national public healthcare routine, for women experiencing infertility. These interventions may help them manage potential mental health problems while pursuing their reproductive goals.

## Methods

### Study design, setting and study population

A cross-sectional study was carried out from October 2023 to April 2024 to assess the mental health status and its associated factors among women with fertility issues attending fertility centers in the Kathmandu Valley. To our knowledge, a disaggregated estimate of infertility specific to the Kathmandu Valley has not been reported in the available literature. However, national-level evidence suggests that infertility affects approximately 10–18% of couples in Nepal [24]. Given that, as per the 2021 national census, the valley is home to over three million people in 899 square kilometers, making it the most densely populated region in Nepal [25,26]. Due to this population density and the concentration of infrastructure and services, Kathmandu Valley hosts the majority of specialized healthcare services in the country, including fertility care. Vatsalya Health Care had the highest patient volume among all fertility centers in Kathmandu valley during the study period; (2) a single-center design was chosen to ensure standardized data collection and minimize heterogeneity in clinical management protocols; and (3) the finite population available at this center was sufficient to achieve the required sample size, as determined by the finite population correction formula. In addition, even though exact patient flow was not provided by fertility centers, Vatsalya is the center with record of more than 5000 success IVF treatment and patients from all over Nepal visited the center [21]. Centers were not included due to feasibility constraints, including access agreements and institutional review board permissions therefore, the study was designed as a single-center cross-sectional as a finite population study. The study population comprised women of reproductive age (18–49 years) diagnosed with primary or secondary infertility who were actively seeking treatment at the selected fertility center during the study period and who provided informed consent to participate. Inclusion criteria were: women aged 18–49 years with a confirmed diagnosis of primary or secondary infertility and willingness to participate in the study. Here, ‘Primary infertility refers to the inability to achieve a pregnancy after 12 or more months of regular unprotected sexual intercourse among women who have never previously conceived. Secondary infertility refers to difficulty conceiving in women who have had at least one prior pregnancy, regardless of its outcome.’ The exclusion criteria were: women with severe psychiatric disorders, those who had undergone infertility treatment elsewhere within the past six months, and women who declined to provide informed consent.

### Sample size, sampling process and study population

The initial sample size was calculated to be 374 using Cochran’s formula for single proportion, assuming a 95% confidence interval (CI), a 5% margin of error, and an estimated proportion (p) of 58%, based on a previous study conducted among infertile women attending a tertiary care hospital in Pakistan [15]. However, 374 samples were not feasible sample size for the researcher to conduct in the limited time frame.

Then a total of 200 eligible clients were identified from the study center’s patient records as having visited during the data collection period (November 2023 to February 2024), representing the entire eligible population within the defined timeframe and thus represented for the purpose of sample size calculation. Given this finite population formula from Cochran, W.G. (1977) [27], the final sample size was recalculated using the finite population correction formula:


$$\begin{array}{c} \text{Sample size }(n)=\frac{n\circ }{\text{1}+\frac{n_{\circ }}{\text{N}}}=\frac{\text{3}\text{7}\text{4}}{\text{1}+\frac{\text{3}\text{7}\text{4}}{\text{200}}}\;=\text{ 130} \end{array}$$

After adding 10% of non-response rate the final sample size for the study was determined to be 143.

From this finite population, 143 participants were then selected using simple random sampling. The ‘RANDBETWEEN’ function in Microsoft Excel was used to generate random numbers for participant selection. Out of the 200 eligible clients identified, 143 were selected through simple random sampling, and all agreed to participate, with no refusals. The remaining 57 individuals were not included due to the random sampling process.

### Ethics statement

All methods of this study were carried out under the Helsinki Declaration of ethical principles for medical research involving human subjects. Ethical approval was taken from the Institutional Review Committee (IRC) of Manmohan Memorial Institute of Health Sciences (NEHCO/IRC/080/048). Permission to conduct the study was obtained from Vatsalya Health Care Pvt. Ltd. for data collection, and from Ishan Children Hospital and IVF Center for pretesting of the data collection instrument. The objectives, methods, and anticipated benefits of the study program were communicated to the participants prior to conducting the research. Written informed consent was obtained from each respondent, without any pressure or inducement of any kind, to ensure the voluntary participation of the respondents. Respondents were ensured voluntary participation and also freedom to withdraw from research at any time as per their comfort. The confidentiality of participants was maintained at every step of data collection, analysis, interpretation, and data dissemination. The personal information of the respondents was not taken to ensure confidentiality throughout the study process.

### Data collection

A structured, validated Nepali version of the DASS-21 (N-DASS21) scale was used to collect the data for this study. DASS-21 is a psychological screening instrument capable of differentiating symptoms of Depression, Anxiety, and Stress (DAS). Depression, anxiety, and stress are three subscales, and there are 7 items in each subscale. DASS 21 is a valid standard scale frequently used by nurses, psychologists, and psychiatrists of Nepal [28]. The DASS-21 is a validated tool and has been used in different groups of the Nepalese population to identify symptoms of depression, anxiety and stress. Cronbach’s alpha coefficient for DASS 21 has been found to range from 0.76 to 0.91. The relevant alphas are: Depression subscale: α = 0.82; Anxiety subscale: α = 0.78; Stress subscale: α = 0.76. [28–30]. (S1 Text) A face-to-face interview with eligible clients was carried out by a trained public health professional.

Each item of the data collection tool was scored on a 4-point Likert scale, which ranges from 0 (did not apply to me at all) to 3 (applied to me very much) [31]. Scores for DAS were calculated by summing the scores for the relevant items and multiplying by two. Depression was assessed using items reflecting dysphoria, hopelessness, devaluation of life, self-deprecation, lack of interest or involvement, anhedonia, and inertia (Items 3, 5, 10, 13, 16, 17, 21). Anxiety was measured through items capturing autonomic arousal, skeletal muscle effects, situational anxiety, and the subjective experience of anxious affect (Items 2, 4, 7, 9, 15, 19, 20). Stress was evaluated based on items indicating levels of chronic nonspecific arousal, difficulty relaxing, nervous arousal, and tendencies to be easily upset, agitated, irritable, over-reactive, or impatient (Items 1, 6, 8, 11, 12, 14, 18). Pretesting of the questionnaire was done among 10% of the sample size among women with fertility issues visiting the Ishan Children Hospital and IVF center in Tokha, Kathmandu.

**Table 1:** The recommended cut-off scores for conventional severity levels.

Severity Level	Depression	Anxiety	Stress
Normal	0-9	0-7	0-14
Mild	10-13	8-9	15-18
Moderate	14-20	10-14	19-25
Severe	21-27	15-19	26-33
Extremely Severe	28+	20+	34+

Source: Note: DASS-21 subscale scores are multiplied by 2 to align with DASS-42 cut-off values (Lovibond & Lovibond, 1995 [29]); the effective score range per subscale is therefore 0–42 (see Table 1) and also been well-established in the psychometric literature by Henry & Crawford (2005) [32].

Along with the major dependent variables, the independent variables were grouped into four domains: (1) socio-demographic factors, including age, ethnicity, religion, educational attainment, family type, age at marriage, duration of marriage, and permanent residence; (2) reproductive and clinical factors, including type of infertility, infertility factor, duration of treatment, treatment cost, and treatment type; (3) psychosocial factors, including feelings of insecurity about not having a child, social stigma, experience of abuse, marital satisfaction, and financial burden; and (4) occupational factors, including employment status and challenges in accessing treatment due to work commitments.

### Data management and analysis

A comprehensive codebook was developed prior to data entry to ensure consistency and standardization of the collected information. The data were initially entered into Microsoft Excel 2019, and once the dataset was cleaned and validated, it was imported into the Statistical Package for Social Sciences (SPSS) version 22.0 for statistical analysis. Descriptive statistics, including frequencies and percentages, were computed for all relevant dependent and independent variables. The independent variables included socio-demographic, reproductive/clinical, psychosocial, and occupational factors, such as age, education, type of infertility, treatment details, social stigma, marital satisfaction, financial burden, and employment status. Most of the independent variables in this study were categorical. Continuous variables, including age, duration of marriage, and treatment duration, were categorized into clinically and epidemiologically meaningful groups prior to analysis as using thresholds drawn from comparable infertility research and clinical practice. Age was categorized as 20–30, 30–40, and 40 + years; duration of marriage as <5, 5-9.99, 10-14.99, and>=15 years; and duration of treatment as <5 and>=5 years.

Bivariate analyses (chi-square tests) were conducted for categorical variables to assess associations between the outcome variable and each explanatory variable. Crude odds ratios (COR) with 95% confidence intervals were estimated from simple (unadjusted) binary logistic regression models for each independent variable. For the logistic regression analyses, each DASS-21 subscale was dichotomized as ‘normal’ (below the mild threshold) versus ‘any level of distress’ (mild, moderate, severe, or extremely severe), based on Lovibond & Lovibond (1995) cut-off values. For depression: normal = 0–9, distress ≥10; for anxiety: normal = 0–7, distress ≥8; for stress: normal = 0–14, distress ≥15. This dichotomization is consistent with the widely adopted binary classification used in infertility and mental health research, as employed by Noorbala et al. (2008) and Bibi et al. (2020). [33,34] Variables with p-value less than 0.25 in bivariate analysis were included in a multivariable binary logistic regression model to assess their independent effects on the outcome, with results presented as adjusted odds ratios (aORs) and 95% confidence intervals. (S1 Data)

To address potential multicollinearity among independent variables included in the multivariable model, the Variance Inflation Factor (VIF) was calculated. A threshold of VIF > 2 was considered indicative of multicollinearity [35], and no multicollinearity was found between the variables. Variables with p-values <0.05 were taken as statistically significant.

## Results

Half of the respondents were aged between 30–40 years (51.7%), followed by those aged 20–30 years (25.9%). In terms of ethnicity, the largest proportion belonged to the Janajati group (33.6%), followed by Chhetri (28.0%) and Brahmin (23.1%). A vast majority of the participants were Hindu (84.6%). In terms of educational attainment, secondary education was most common (41.3%), followed by a university degree (32.9%), a postgraduate degree (12.6%) and more than half (56.6%) of participants lived in a joint family. The median age at marriage was 24 years (IQR ± 5). More than half (55.9%) were married at age 24 or older. Regarding the duration of marriage, one-third (33.6%) had been married for less than 5 years whereas almost one out of four (23.8%) were married for 15 years or more. In terms of permanent residence, 62.9% were from outside the Kathmandu Valley, 34.3% resided within the valley, and a small proportion (2.8%) reported their permanent address as being outside Nepal (Table 2).

**Table 2:** Socio-demographic characteristics of the respondents (n = 143).

Variables	Frequency (n = 143)	Percentage
**Age in years (Min-23, Max-50)**		
20-30	37	25.9
30-40	74	51.7
40+	31	22.3
**Ethnicity**		
Janajati	48	33.6
Chhetri	40	28.0
Brahmin	33	23.1
Madhesi	12	8.4
Dalit	8	5.6
Others	2	1.4
**Religion**		
Hindu	121	84.6
Buddhism	11	7.7
Christianity	9	6.3
Muslim and other	2	1.4
**Highest level of education**		
No education (never been to school)	1	0.7
Basic Education	18	12.6
Secondary Education	59	41.3
University degree	47	32.9
Post graduate degree	18	12.6
**Family type**		
Nuclear family	62	43.4
Joint family	81	56.6
**Age at marriage (in years) (Median ± IQR: 24 ± 5)**		
<24	63	44.1
≥24	80	55.9
**Duration of marriage (in years)**		
<5	48	33.6
5-9.99	40	28.0
10-14.99	21	14.7
≥15	34	23.8
**Permanent Address**		
Inside valley	49	34.3
Outside valley	90	62.9
Out of Nepal	4	2.8

Among the 143 women interviewed, almost half were (44.8%) working. Among the women working (n = 64), the highest percentage (70.3%) were involved in Government/private service. More than one-third of the respondents were unemployed (38.5%), and 16.8% of women had discontinued employment primarily to dedicate time to fertility treatment. Among working women (n = 64), 45.3% reported very difficult and 23.4% reported very great difficulty in attending the clinic due to work commitments; combined, more than two-thirds (68.7%) experienced some form of access challenge related to their employment. Among them, 56.5% managed their attendance by taking daily leave from work. The median monthly family income was NRS 60,000 (IQR + /-50,000), equivalent to approximately USD 450 -- above the national minimum wage, though modest by urban Kathmandu standards. More than half of respondents (54.5%) reported a family income at or above this figure. (Table 3).

**Table 3:** Socio-economic characteristics of the respondents (n = 143).

Variables	Frequency (n = 143)	Percentage
**Working status**		
Yes	64	44.8
No	55	38.5
Left job due to treatment	24	16.8
**Types of Occupation (n = 64)**		
Government/private service oriented	45	70.3
Self-employed (business/freelance/entrepreneur)	15	23.4
Worker/laborer/daily wages	4	6.3
**Time off work to visit clinic (n = 64)**		
Easy	7	10.9
Neither easy nor difficult	13	20.3
Difficult	29	45.3
Very difficult	15	23.4
**Managing treatment for work (n = 64)**		
Daily basis leave from work	36	56.5
Appointing staff/help from families in own business	11	17.2
Complete leave for treatment	10	15.6
Manage working hours accordingly	7	10.9
**Total monthly income (Median ± IQR) (60,000 ± 50,000) (in NRS)**		
<60,000	65	45.5
≥60,000	78	54.5

More than three-quarters of the respondents (77.6%) were satisfied with their marital life. Among the 7 women who reported marital dissatisfaction, 2 cited their husband’s alcohol use and 2 cited the absence of a child as a key reason for dissatisfaction. Having child was essential to most (86.7%) of the women. More than half of the respondents (58.7%) had never conceived in their lifetime, and more than two-thirds of respondents (72%) were insecure about not having a child. More than three-fourths of the respondents (76.2%) had not noticed any changes in families’ behavior after the fertility issues. Among the respondents (n = 34) who had faced changes in their families’ behaviors, 41.18% were accused of not having an heir of the family, and 17.7% each were accused of being ill and faced different arguments, respectively. Whereas 14.7% of respondents’ families asked their husbands to remarry to have a child, and the remaining 8.9% had lost their importance and support from family as in earlier days. Most families were either neutral (40.6%) or very supportive (39.2%), indicating generally moderate to strong family support during treatment. (Table 4).

**Table 4:** Family level characteristics of the respondents (n = 143).

Variables	Frequency (n = 143)	Percentage
**Marital Satisfaction**		
Yes	111	77.6
No	7	4.9
Neutral	25	17.5
**Reasons for dissatisfaction (n = 7)**		
Husband drinks a lot	2	28.6
No child	2	28.6
No support from family	1	14.3
Divorce and lost child in accident	1	14.3
Violence from husband’s family	1	14.3
**Parity**		
Never Conceived	84	58.7
Conceived Earlier but no live children	45	31.5
Had live children earlier but difficulty in conceiving now	13	9.1
Others (Adoption)	1	0.7
**Importance to have a child**		
Very Important	124	86.7
Important	19	13.3
**Insecure about not having a child**		
Yes	103	72.0
No	40	28.0
**Change in families’ behaviors**		
Yes	34	23.8
No	109	76.2
**Types of changes in families behaviors (n = 34)**		
Accused of not having the heir of the family	14	41.2
Accused of being ill	6	17.7
Often arguments and no support	6	17.7
Asked husband to remarry	5	14.7
Not giving much importance as earlier	3	8.8
**Support of family (in-laws) for the treatment**		
Not supportive at all	2	1.4
Not so supportive	6	4.2
Neither supportive nor unsupportive	58	40.6
Supportive	21	14.7
Very supportive	56	39.2

Half of the respondents (50.3%) had faced social stigmas during their lifetime due to fertility issues. Among women who reported experiencing social stigma (n = 71), the most common form was being subjected to character judgments and derogatory remarks (23 out of 71, 32.4%), followed by 21.1% of women accused of being ‘*Bajh*’ and also 8.5% of respondents’ husbands were asked to remarry by the relatives and society.

Similarly, more than two-thirds of the respondents (70.6%) were involved in social activities. Among women who were not involved in social activities (n = 42), the most frequently cited barrier was lack of time owing to household and work responsibilities (16/42, 38.1%), followed by fear of judgment or social stress (12/42, 28.6%), and lack of interest in participating (8/42, 19.1%). Well above three-fourth of the respondents (78.3%) felt comfortable sharing their personal feelings with anyone. Regarding the abuse faced due to fertility issue during their lifetime, 13.3% (n = 19) of the women had positive response. Among those who reported abuse (n = 19), the most common perpetrators were members of the broader community or society (84.2%, n = 16) (e.g., neighbors, close friends, community members), followed by relatives (47.4%), and in-laws or other family members (26.3%). Among total self-reported abuse (n = 19), around three-fourths of respondents (73.7%) faced both verbal and emotional abuse, 15.7% verbal abuse only (Table 5).

**Table 5:** Psychological and social characteristics of the respondents (n = 143).

Variables	Frequency (n = 143)	Percentage
**Social stigmas faced due to infertility**		
Yes	71	50.3
No	72	49.7
**Types of Social stigmas faced (n = 71)**		
Judgments on personality and negative comments	23	32.4
Accused of being ‘*bajh*’	15	21.1
Accused of being career-oriented and married late	9	12.7
Still not conceived after a long duration of marriage	8	11.3
Accused of being incomplete, wrong or ‘*papi’*	6	8.5
Second marriage suggestions to husband	6	8.5
Stereotypical judgments on IVF	4	5.7
**Involved in social activities**		
Yes	101	70.6
No	42	29.4
**Reasons for not being involved in social activities (n = 42)**		
No free time from household chores and office	16	38.1
Fear of judgments and stress	12	28.6
Not interested in even participating	8	19.1
Always asks for child	4	9.5
Jealous of other women and kids	2	4.8
**Share feelings with anyone**		
Yes	112	78.3
No	31	21.7
**Reasons for not sharing feelings with anyone (n = 31)**		
Not comfortable to share personal stuffs	19	61.3
Fear of judgments from others	8	25.8
Male factor infertility cases stereotypes	4	12.9
**Abuse due to infertility issues**		
Yes	19	13.3
No	124	86.7
Reported abusers* (n = 19)		
Society	16	84.2
relatives	9	47.8
In-laws	5	26.2
Husband and other family members	5	26.2
Husband	1	5.3
Others	0	0
**Types of abuses (n = 19)**		
Verbal and emotional	14	73.7
Verbal	3	15.7
Emotional	1	5.3
Sometimes physical	1	5.3

Multiple response*.

*Note: ‘Bajh’ is a Nepali/local vernacular term used to describe a woman who is unable to conceive, often used in a stigmatizing sense within certain communities in Nepal. It is culturally equivalent to the label of ‘barren.’*

Among 143 respondents, the vast majority of the respondents (90.2%) had primary infertility issues. More than one-fifth of the respondents (42.7%) had fertility issues for more than 5 years. Among all participants, the female infertility factor was most prevalent identified in 62.9% of the cases, followed by unexplained infertility (26.6%), and male factor infertility in 10.5% cases. Half of the respondents (50.3%) had also undergone treatment before the specific period of time of data collection, with almost one-third (31.9%) got treatment already for 2 times. More than half of the respondents (51.7%) had IVF from couple’s own egg and sperm and more than half (59.4%) of them were seeking treatment for less than 2 years.

More than half of the respondents (56.6%) shared the infertility treatment procedure with family members. Among 62 respondents who hadn’t shared it with family, two-fifth of them (40.3%) had fear of judgments and unacceptance of infertility treatment due to different stereotypes in rural areas. More than half of the respondents (56.6%) had treatment cost less than NRS 5 lakhs, and nearly half of the respondents (49.7%) had a financial burden. Among those with a financial burden (n = 71), more than two-thirds (66.2%) reported delays in treatment initiation and continuation due to higher treatment costs and financial constraints. Additionally, more than a third had plans to sell land or other properties to fund future treatment costs (Table 6).

**Table 6:** Treatment related characteristics of the respondents (n = 143).

Variables	Frequency (n = 143)	Percentage
**Type of infertility**		
Primary	129	90.2
Secondary	14	9.8
**Duration of infertility**		
< 2 years	50	35
2-5 years	32	22.4
> 5 years	61	42.7
**Factor of infertility**		
Female	90	62.9
Unexplained	38	26.6
Male	15	10.5
**Treatment procedure (Recent)**		
Timed intercourse/Ovulation induction	1	0.7
Intrauterine insemination (husband sperm)	8	5.6
Intrauterine insemination (Donor sperm)	3	2.1
In-vitro fertilization (Couple)	74	51.7
In-vitro fertilization (Donor sperm)	10	7
In-vitro fertilization (egg donation)	45	31.5
Intra-cytoplasmic sperm injection (husband sperm)	1	0.7
Intra-cytoplasmic sperm injection (donor sperm)	1	0.7
**Any treatment before**		
Yes	72	50.3
No	71	49.7
**Treatment times (before) n = 72**		
once	22	30.6
2 times	23	31.9
3 times	15	20.8
4 times	5	6.9
5 times	6	8.3
6 times	1	1.4
**Duration of getting treatment (all)**		
< 2 years	85	59.4
2-5 years	26	18.2
more than 5years	32	22.4
**Infertility shared with family**		
Yes	81	56.6
No	62	43.4
**Reasons for not sharing with family (n = 62)**		
Fear of judgments and unacceptance due to stereotypical thoughts in rural areas	25	40.3
Fear of in-laws and other family members	23	37.1
Fear of treatment and its negative results	9	14.5
Secondary infertility	3	4.8
Male infertility factor	2	3.2
**Thought of sharing to family in the future (n = 62)**		
Yes	2	3.2
No	60	96.8
**Treatment cost (till now)**		
<500,000 NRS	81	56.6
≥500,000 NRS	62	43.4
**Financial Burden**		
Yes	71	49.7
No	72	50.3
**Delay in treatment (n = 71)**		
Yes	47	66.2
No	24	33.8
**Managing the cost (n = 71)**		
All savings, salary and house rents	31	43.7
Complete loans	20	28.2
Selling properties, gold	12	16.9
Borrowing from friends, closed ones	8	11.3
**Future financial treatment plans (n = 71)**		
Selling properties, lands	27	38.0
Loans to be added	18	25.4
Savings and salaries	12	16.9
Business, rents from house	9	12.7
Ask to families	5	7.0

A high proportion of respondents reported some level of psychological distress across all three dimensions of the DASS-21. Among the 143 respondents, 14% experienced severe stress, and 1.4% reported extremely severe stress. Regarding anxiety, 44.1% of respondents had moderate anxiety, followed by 14.7% with severe and 11.2% with extremely severe anxiety. In terms of depression, 19.6% of respondents reported mild depression, while 2.8% and 0.7% experienced severe and extremely severe depression, respectively. Overall, more than four-fifths of respondents (82.5%) exhibited some level of anxiety, followed by 51.7% with stress and 35.0% with some degree of depression (Table 7).

**Table 7:** DASS Tool Scale Analysis.

Variables	Frequency (n = 143)	Percentage
**Stress Level**		
Normal	69	48.3
Mild	31	21.7
Moderate	21	14.7
Severe	20	14
Extremely Severe	2	1.4
**Anxiety level**		
Normal	25	17.5
Mild	18	12.6
Moderate	63	44.1
Severe	21	14.7
Extremely Severe	16	11.2
**Depression level**		
Normal	93	65
Mild	28	19.6
Moderate	17	11.9
Severe	4	2.8
Extremely Severe	1	0.7

Several sociodemographic, psychosocial, and clinical variables were found to be significantly associated with depression, anxiety, and stress in the multivariable logistic regression analyses. The odds of having stress among women facing abuse was 12.76 times higher (AOR = 12.76, 95% CI = 1.38-118.13) than women not facing the abuse during their lifetime, adjusting all other independent variables and similarly odds of having stress among women who were insecure about not having a child was 3.19 times (AOR = 3.19, 95% CI = 1.98-10.39) than those who weren’t insecure. Likewise, the odds of having stress among women facing social stigmas was 2.91 times (AOR = 2.91, 95% CI = 0.93-9.09) as compared to the women not facing the social stigmas. (Table 8).

**Table 8:** Multiple Logistics Regression between Stress and associated factors.

Characteristics	COR	95% CI	p-value	AOR	95% CI	p-value
**Permanent Address**						
Inside valley	Ref			Ref		
Outside the valley	2.23	1.10-4.51	0.026	2.13	0.81-5.60	0.128
**Type of family**						
Nuclear family	Ref			Ref		
Joint family	2.83	1.45-5.72	0.002	1.81	0.71-4.67	0.217
**Marital satisfaction**						
Yes	Ref			Ref		
No and neutral	2.50	1.08-5.76	0.032	1.14	0.36-3.57	0.826
**Parity**						
Never conceived	3.50	0.91-13.44	0.68	1.76	0.05-59.89	0.754
Conceived but no live children	7.33	1.77-30.31	0.006	2.84	0.09-91.19	0.556
Hard to conceive now	Ref			Ref		
**Insecure about not having a child**						
No	Ref					
Yes	7.14	2.98-17.08	<0.001	3.19	1.98-10.39	0.044*
**Changes in family’s behavior**						
No	Ref			Ref		
Yes	4.13	1.72-9.94	0.002	1.36	0.35-5.33	0.656
**Family support**						
Not supportive	2.46	1.25-4.84	0.009	0.98	0.35-2.76	0.97
Supportive	Ref			Ref		
**Social Stigmas**						
No	Ref			Ref		
Yes	6.22	3.01-12.86	<0.001	2.91	0.93-9.09	0.046*
**Abuse**						
No	Ref			Ref		
Yes	21.86	2.83-168.85	0.003	12.76	1.38-118.10	0.025*
**Type of infertility**						
Primary	4.49	1.20-16.85	0.026	1.23	0.04-39.49	0.905
Secondary	Ref			Ref		
**Duration of treatment**						
<5 years	Ref			Ref		
≥5 years	2.50	1.08-5.76	0.032	0.84	0.23-3.05	0.791
**Been in treatment before**						
No	Ref			Ref		
Yes	2.72	1.38-5.35	0.004	0.88	0.29-2.64	0.82
**Treatment cost**						
<5,00,000 NRS	Ref			Ref		
≥5,00,000 NRS	3.22	1.61-6.44	0.001	1.99	0.78-5.07	0.149
**Financial burden**						
No	Ref			Ref		
Yes	2.57	1.31-5.04	0.006	0.55	0.18-1.66	0.291

*Indicates statistically significant at p-value < 0.05.

The odds of having anxiety among Relative to women who were currently experiencing difficulty conceiving (the reference group), those who had never conceived had 2.56 times higher odds of anxiety (AOR = 2.56, 95% CI = 1.65-36.09, p = 0.020), and those who had previously conceived but had no living children had 4.86 times higher odds (AOR = 4.86, 95% CI = 2.61-90.76, p = 0.009).Similarly, the odds of having anxiety among women facing financial burden was 4.78 times (AOR = 4.78, 95% CI = 1.05-21.89) higher than women not facing financial burden, adjusting for all other variables (Table 9).

**Table 9:** Multiple Logistic Regression between Anxiety and associated factors.

Characteristics	COR	95% CI	p-value	AOR	95% CI	p-value
**Level of education**						
School education or below	3.10	1.24-7.75	0.016	1.41	0.37-5.35	0.617
University education	Ref			Ref		
**Type of family**						
Nuclear family	Ref			Ref		
Joint family	4.33	1.67-11.18	0.003	3.02	0.84-10.89	0.092
**Employment status**						
No	3.21	1.28-8.04	0.013	1.10	0.34-3.57	0.87
Yes	Ref			Ref		
**Parity**						
Never conceived	2.19	0.65-7.39	0.206	2.56	1.65-36.09	0.02*
Conceived but no live children	7.78	1.57-38.61	0.012	4.86	2.61-90.76	0.009*
Hard to conceive now	Ref			Ref		
**Insecure about not having a child**						
No	Ref			Ref		
Yes	2.97	1.22-7.24	0.017	1.05	0.31-3.60	0.936
**Social Stigmas**						
No	Ref			Ref		
Yes	5.08	1.79-14.44	0.002	1.55	0.39-6.17	0.043*
**Been in treatment before**						
Yes	2.52	1.01-6.29	0.048	0.76	0.20-2.84	0.686
No	Ref			Ref		
**Financial burden**						
Yes	6.90	2.23-21.34	0.001	4.78	1.05-21.89	0.044*
No	Ref			Ref		

*Indicates statistically significant at p-value<0.05.

The odds of having depression among non-Hindu women were 7.61 times (AOR = 7.61, 95% CI = 1.73-33.49) those of Hindu women. The odds of having depression among women who were not satisfied with their married life was 4.30 times (AOR = 4.30, 95% CI = 1.12-16.50) that of satisfied women. Similarly, the odds of having depression among women having female factors of infertility were 14.14 times (AOR = 14.14, 95% CI = 1.57-127.40) higher than for those with an unexplained factor of infertility. The odds of having depression among women having treatment duration more than 5 years were 3.34 times (AOR = 3.34, 95% CI = 0.82-13.61) higher than women having treatment duration less than this. Likewise, the odds of having depression among women who had treatment costing more than five lakhs was 2.71 times (AOR = 2.71, 95% CI = 0.21-26.74) higher than women having treatment costing less than that, adjusting other variables (Table 10).

**Table 10:** Multiple Logistics Regression between Depression and associated factors.

Characteristics	COR	95% CI	p-value	AOR	95% CI	p-value
**Age (in years)**						
20-30	Ref			Ref		
30-40	4.12	1.44-11.81	0.008	3.81	0.51-28.66	0.193
40+	6.40	1.99-20.62	0.002	5.63	0.51-62.20	0.159
**Religion**						
Hindu	Ref			Ref		
Non-Hindu	3.28	1.29-8.34	0.013	7.61	1.73-33.49	0.007*
**Age at marriage (in years)**						
<24	1.86	0.93-3.73	0.081	2.26	0.53-9.67	0.271
≥24	Ref			Ref		
**Duration of marriage (in years)**						
<5	Ref			Ref		
5-9.99	3.37	1.14-9.94	0.028	2.24	0.43-11.68	0.337
10-14.99	9.33	2.77-31.49	<0.001	1.43	0.17-12.41	0.746
≥15	8.87	2.98-26.40	<0.001	0.82	0.10-6.97	0.855
**Level of education**						
School education or below	3.12	1.49-6.55	0.003	1.28	0.29-5.66	0.743
University education	Ref			Ref		
**Type of family**						
Nuclear family	Ref			Ref		
Joint family	2.38	1.15-4.94	0.019	2.79	0.72-10.76	0.137
**Monthly family income**						
<60,000 NRS	2.07	1.02-4.22	0.045	0.46	0.11-1.95	0.292
≥60,000 NRS	Ref			Ref		
**Marital satisfaction**						
Yes	Ref			Ref		
No and neutral	5.40	2.32-12.55	<0.001	4.30	1.12-16.50	0.033*
**Insecure about not having a child**						
Yes	7.26	2.41-21.899	<0.001	2.91	0.51-16.54	0.229
No	Ref			Ref		
**Family support**						
Supportive	Ref			Ref		
Not supportive	2.38	1.18-4.80	0.016	1.05	0.29-3.85	0.938
**Social Stigmas**						
Yes	4.26	2.02-8.99	<0.001	0.77	0.18-3.32	0.729
No	Ref			Ref		
**Shared feelings with anyone**						
Yes	Ref			Ref		
No	4.14	1.80-9.52	0.001	2.25	0.57-8.90	0.247
**Abuse**						
Yes	5.10	1.80-14.43	0.002	2.63	0.55-12.54	0.226
No	Ref			Ref		
**Duration of infertility**						
<2 years	Ref			Ref		
2-5 years	1.21	0.38-3.89	0.747	0.86	0.15-5.14	0.87
≥5 years	7.56	3.04-18.82	<0.001	2.98	0.43-20.85	0.272
**Factor of infertility**						
Female	3.90	1.48-10.25	0.006	14.14	1.57-127.40	0.018*
Male	3.56	0.92-13.74	0.066	3.92	0.77-20.01	0.1
Unexplained	Ref			Ref		
**Duration of treatment**						
<5 years	Ref			Ref		
≥5 years	9.81	3.94-24.43	<0.001	3.34	0.82-13.61	0.042*
**Been in treatment before**						
Yes	5.50	2.53-11.92	<0.001	1.96	0.56-6.82	0.29
No	Ref			Ref		
**Treatment cost**						
<500,000 NRS	Ref			Ref		
≥500,000 NRS	2.86	1.41-5.81	0.004	2.71	0.22-26.74	0.023*
**Financial burden**						
Yes	2.81	1.37-5.75	0.005	0.66	0.18-2.45	0.53
No	Ref			Ref		

*Indicates statistically significant at p-value<0.05.

‘NRS 500,000 (five lakhs; one lakh = 100,000; approximately USD 3,700)

## Discussion

The results of the current study assessed the multifaceted mental health status and associated experiences of infertile Nepalese women. More than 8 out of 10 respondents (82.5%) had some form of anxiety, more than half (51.2%) had some form of stress, and 35.0% of women had some form of depression, as found in the present study. A study conducted in Iran among infertile women receiving treatment at a clinical setting also reported a high prevalence of depression (40.8%) and anxiety (86.8%), comparable to the present study. [13]. The alignment in results may be explained by the shared clinical context and extended duration of infertility treatment in both cohorts, which is known to compound psychological distress. In contrast, a study conducted among infertile women (20–45 years of age) in a tertiary hospital in Pakistan reported higher prevalences of depression (58.2%), anxiety (57.3%), and stress (50.0%) associated with type of infertility [15]. The difference in psychological distress prevalence between Nepal and Pakistan may reflect broader contextual differences – including healthcare infrastructure, treatment affordability, insurance systems, and cultural expectations around motherhood.

This study found a significant association between anxiety and parity: women who had never conceived had 2.56 times higher odds of anxiety (AOR = 2.56, 95% CI = 1.65-36.09, p = 0.020), and those who had previously conceived but had no living children had 4.86 times higher odds (AOR = 4.86, 95% CI = 2.61-90.76, p = 0.009), relative to women who were currently experiencing difficulty conceiving. This association likely reflects the compounding uncertainty and grief experienced by women who have never had a positive pregnancy experience. Similar findings were found in a study assessing depression, anxiety, and stress among infertile women in Pakistan, where a significant relationship between DASS scores and parity was found (p = 0.049) [13]. The study in Pakistan was also conducted in similar settings among infertile women getting treatment, and where motherhood is the sole way for women to advance in life in both of the countries, which may have added on greater anxiety in women with parity factor. Similarly, participants experiencing financial burden had significantly higher odds of anxiety compared to those without financial burden (AOR = 4.78; 95% CI: 1.05–21.89; *p* = 0.044) in this study. The absence of insurance coverage for IVF treatment in Nepal likely increases financial strain among women already navigating a prolonged and emotionally taxing process, contributing to the higher anxiety observed in this study.

This study also identified women facing social stigmas were 1.55 times (AOR = 1.55, 95% CI = 0.389-6.165) more likely to have anxiety than women not facing the social stigmas. While in a similar study conducted in Nigeria, social stigma was associated with higher rates of anxiety and depression [36]. Experiencing stigmatizing behavior (such as humiliation, discrimination, and devaluation) was associated with a threefold higher rate of depression and anxiety [13]. Similarly, Chinese women with infertility reported discrimination, shame, and reproductive pressures associated with depression [37]. Whereas, in the study of Assessment of depression, anxiety and stress among infertile women in Pakistan, emotional strain of infertile women from in-laws was also associated with DASS (p = 0.001) [13]. The study contradicts the findings of this study as family and husband support in treatment may be less in Muslim communities in Pakistan than in Nepal due to existing stereotypes and the status of women in Muslim countries.

The patriarchal family structures prevalent in this study region, combined with the strong cultural emphasis on motherhood, often place disproportionate blame on women for childlessness, further exacerbating their psychological vulnerability. Infertility often leads to feelings of inadequacy; in this study, women who were insecure about not having a child were 3.19 times (AOR = 3.19, 95% CI = 1.98-10.39) more likely to have stress than those who were not insecure. Also, the participants experiencing social stigma had higher odds of stress compared to those without stigma (AOR = 2.91; 95% CI: 0.93–9.09; *p* = 0.046). Similarly, those who experienced abuse had significantly higher odds of stress (AOR = 12.76; 95% CI: 1.38–118.10; *p* = 0.025). Similar findings were seen in research in Iran, where infertility often leads to feelings of inadequacy, isolation, and disrupted self-identity, facing social stigmas and abuses which, in turn, can trigger a cascade of psychological responses [38]. This suggests infertility has created the feeling of inadequacy and insecurity, and jealousy among women all around the world, which has affected the psychological state of women battling with it, also they are further intensified by external traumas like stigmas and abuses. This needs appropriate counseling techniques and social awareness campaigns by the government and/or policymakers to reduce the burden of poor mental health among infertile women, thus ensuring a better quality of life.

Similarly, this study identified women having female factors of infertility were 14.14 times (AOR = 14.14, 95% CI = 1.57-127.40) more likely to have depression than those with an unexplained factor of infertility. This contradicts the findings of a cross-sectional study that was conducted in Tehran, Iran, where participants who had both/unknown causes of infertility were more likely to have poor well-being than men and women who had male and female factor infertility, respectively (OR = 1.54, 95% CI: 0.99–2.40), although this difference was not statistically significant (P = 0.053) [39]. In Nepal’s largely patriarchal social structure, women are often held personally responsible for infertility regardless of where the medical cause lies. When a female factor is identified, this pre-existing social blame may intensify – the woman is seen not just as failing to conceive, but as being confirmed as the source of the problem. This dynamic, we believe, helps explain why female factor infertility showed such a strong association with depression in our sample.

This study also showed depression was significantly associated with duration of treatment, where women having treatment for more than 5 years were 3.34 times (AOR = 3.34, 95% CI = 0.82-13.61) more likely to have depression than women having treatment for less than 5 years. Similar findings were seen in a study conducted in Iran where anxiety and depression were most common after 4–6 years of infertility treatment (p = 0.004) and severe depression could be found in those who had infertility treatment for 7–9 years [13]. In the similar study conducted by another Iranian university with 300 infertile women in three infertility centers, the mean psychological well-being score (PWB) in women who underwent IVF more than once was lower than the subjects who underwent IVF only once. Also, the mean PWB score in women who had more than once failed IVF pregnancies was lower than in subjects who had not had pregnancies by IVF. Also, the results of a qualitative study showed infertile women who receive IVF treatment because of receiving more hard treatment, failure treatment or failed IVF pregnancies feel out of control over their lives, which in turn reduces PWB [40].The prolonged duration and intensive nature of fertility treatment, including multiple failed in vitro fertilization (IVF) cycles, is known to contribute substantially to the development and persistence of depressive symptoms, consistent with findings from both the present study and the Iranian cohort.

Similarly, this study depicts the financial burden (AOR = 4.78, 95% CI = 1.05-21.89) and marital satisfaction as significant (AOR = 4.30, 95% CI = 1.12-16.50) with depression level which was similar to the findings from a study in Iran in which among infertile women, age, social and sexual concern, marital relationship stress and financial stress were significantly related to distress [15]. This suggests high IVF treatment costs and no insurance system coverage for IVF as well as marital dissatisfaction, may have increased insecurity among women for not being able to have a child even after a long duration of marriage

### Limitations

Firstly, as the study was conducted in a single center with a finite and non-representative population, the findings may have limited generalizability beyond the study setting. However, the selected center has the highest patient volume in Kathmandu district and may reasonably reflect the characteristics of women attending high-volume urban fertility facilities; however, the experiences of women receiving care at other centers, particularly smaller, private, or rural facilities may differ. The exclusion of other centers due to logistical and regulatory constraints may restrict broader population-level inference, and the results should be interpreted within the context of a single-center cross-sectional design. Secondly, although regression models were specified based on theoretical and empirical considerations, the relatively small sample size and limited number of outcome events may increase the risk of model overfitting and reduced estimate precision. And, several associations’ yielded confidence intervals that are wide and include the null value (1.0), which is likely a consequence of limited sample size and sparse data in some subcategories and therefore the strength and stability of these associations should be interpreted with caution. Third, the cross-sectional design precludes any inference of causality or temporal relationships between exposures and outcomes. Fourth, data were based on self-reported measures, which may be subject to recall and social desirability bias. Finally, the possibility of residual or unmeasured confounding cannot be completely excluded. Future research employing multi-center studies across diverse settings and longitudinal study would help strengthen external validity, casual interpretation and improve the generalizability of the results. However, this study highlights the high burden of psychological distress among women seeking infertility treatment in a high-volume urban fertility center in Nepal, underscoring the need for routine mental health screening and integrated psychosocial support in infertility care programs.

## Conclusion

The majority of the women had anxiety, while half of the women had stress and nearly one-third of the women had some level of depression in this study. It also indicate that within the study sample, specifically anxiety compared to women with secondary infertility as determined by multivariable logistic regression, parity emerged as a significant predictor of anxiety: women who had never conceived, and those who had previously conceived but had no living children, both showed significantly higher odds of anxiety than women currently struggling to conceive. This finding points to the particular psychological weight carried by women with no prior pregnancy experience, and underscores the need for targeted mental health support for this subgroup within fertility care settings.. The findings showed the higher association between the mental health status of women with fertility issues with mostly social and treatment-related factors like insecurity, social stigma, abuse, parity, and duration of treatment, factor of infertility, treatment cost and financial burden. These social and treatment-related factors create a complex interplay that impacts the mental health status of women facing fertility issues. Recognizing and addressing these factors within fertility care settings is crucial for providing comprehensive support and improving the overall well-being of women navigating infertility challenges. The findings underscore the importance of providing holistic support and tailored interventions to address the mental health needs of women experiencing fertility challenges, thereby enhancing their overall psychological well-being within the context of fertility care.

## Supporting information

S1 Text Study Tool. (DOCX)

S1 Data Data Set of the Study. (SAV)

## Abbreviations

AOR: Adjusted Odds Ratio
CI: Confidence Interval
COR: Crude Odds Ratio
DASS: Depression, Anxiety, and Stress Scale
IQR: Inter Quartile Range
IVF: In Vitro Fertilization
NRS: Nepalese Rupees
PWB: Psychological Well-being
SPSS: Statistical Package for Social Sciences
VIF: Variance Inflation Factor
WHO: World Health Organization
