# Supplementary material for: Mental health status and its associated factors among women with fertility issues visiting fertility center in Kathmandu Valley: A cross-sectional study
Source: PLOS Glob Public Health. 2026 Aug 27;6(8):e0005487. doi: 10.1371/journal.pgph.0005487 (PMC13521362; doi:10.1371/journal.pgph.0005487)
Supplement: Supplementary file 1 [file pgph.0005487.s001.docx]

| **ANNEX 1- SELF REPORTED BY RESPONDENTS** | | | |
| --- | --- | --- | --- |
| **Q. N** | **Questions** | **Response** | **Skipping pattern** |
| **Socio-demographic questionnaire** | | |  |
| 1 | Age (Complete) | Answer: …………. |  |
| 2 | Ethnicity | 1. Brahmin 2. Chhetri 3. Madhesi 4. Dalit 5. Janajati 6. Others |  |
| 3 | Religion | 1. Hindu 2. Buddhism 3. Christianity 4. Muslim 5. Others |  |
| 4 | Permanent Address | District ……………...  Metropolitan/Sub-metropolitan/ Municipality (Urban/ rural |  |
| 5 | Temporary Address | District ……………...  Metropolitan/Sub-metropolitan/ Municipality (Urban/ rural) |  |
| 6 | Age at marriage (complete years) | Answer……… |  |
| 7 | Duration of marriage | Answer………. |  |
| 8 | Highest level of education | 1. No Education (never been to school) 2. Literate (never been to school but can read and write simple Nepali language) 3. Basic education level (1-8) 4. Secondary Education level (9-12) 5. University Degree (Graduate) 6. Post graduate degree |  |
| 9 | What kind of family are you living in? | 1. Nuclear family 2. Joint family |  |
| **Socio-economic factors questionnaire** | | |  |
| 10 | Are you currently working? | 1. Yes 2. No | If No go to Q.N 1.14 |
| 11 | What type of occupation are you involved in? | 1. Worker/ Laborer/ Daily wages 2. Government/ private service oriented 3. Self-employed   (Business/Freelance/ Entrepreneur) |  |
| 12 | How easy is it for you to get time off from work to visit the clinic for treatment? | 1.Very easy  2.Easy  3.Neither Easy nor difficult  4.Difficult  5.Very difficult |  |
| 13 | How are you managing your work during the treatment process? | Answer: …………………………… |  |
| 14 | What is the total monthly income of the family? | Answer: ……………………………………. |  |
| 15 | Have you shared your infertility issues with family or not? | 1.Yes  2.No  Ifno why?................................................. |  |
| 16 | What is the total estimated cost of treatment till now? | Answer…………... |  |
| 17 | What is the source of your financial arrangement for your treatment? | 1. Oneself 2. Couples 3. Husband 4. Family (Husband’s side) 5. Family (Wife’s side) 6. Others |  |
| 18 | Does your treatment cost bring financial burden to your family? | 1. Yes 2. No | If No go to Q.N 1.22 |
| 19 | Does this financial burden ever delay your treatment procedure? | 1. Yes 2. No |  |
| 20 | So, how are you managing the cost of treatment till now? | Answer…………………………………………………………………… |  |
| 21 | What are your future financial plans to cover the cost of treatment? | Answer………………………………………………………………… |  |
| **Social factors questionnaire** | | |  |
| 22 | Are you satisfied with your married life? | Answer:   1. Yes 2. Neutral 3. No   If no, why?  …………………………………  If yes, why?  ……………………………………. |  |
| 23 | Status of parity | 1. Never conceived 2. Conceived earlier but no live children 3. Had live children earlier but hard to conceive now 4. Others……. |  |
| 24 | How important it is for you to have a child? | 1. Very Important 2. Important 3. Not so important |  |
| 25 | Have you ever felt insecure of not having a child? | 1. Yes 2. No |  |
| 26 | Does this problem have brought the change in your husband’s behaviors? | 1. Yes 2. No   If yes what are the changes?........................ |  |
| 27 | Does this problem have brought the change in your family behaviors? | 1. Yes 2. No   If yes what are the changes?........................ |  |
| 28 | How supportive is your family (In laws) during the infertility treatment process? | 1. Not supportive at all 2. Not so supportive 3. Neither supportive nor non-supportive 4. Supportive 5. Very supportive |  |
| 29 | How supportive is your husband during the infertility treatment process? | 1. Not supportive at all 2. Not so supportive 3. Neither supportive nor non-supportive 4. Supportive 5. Very supportive |  |
| 30 | Do anyone accompany you to the hospital for the treatment? | 1.Yes  2. No  3. Visits when called by the doctor   1. No11111 | If No go to Q.N 32 |
| 31 | If yes who accompany you more frequently? **(Multiple response)** | 1. Husband 2. In-laws 3. Parents 4. Friends 5. Relatives 6. Others (Please specify) |  |
| 32 | Have you faced any social stigmas due to the infertility throughout your life? | 1. Yes 2. No   If yes…, what are they?  …………………………………… |  |
| 33 | Are you actively involved in social activities like social gatherings, social women groups etc.? | 1.Yes  2.No  If no …why?  …………………………………… |  |
| 34 | Do you share your feelings with anyone? | 1. Yes 2. No   If no, why are you not willing to share? ……………………… | If No go to Q.N 36 |
| 35 | If yes to whom you share your feelings most frequently?  **(Multiple response)** | 1. Husband 2. Family members 3. Friends 4. Relatives 5. Social women groups 6. Others (please specify) |  |
| 36 | If you haven’t shared till date to the family, have you thought of sharing your feelings anytime in the future? | 1. Yes 2. No   If no why?......................... |  |
| 37 | Have you ever faced any abuse due to infertility issues? | 1. Yes 2. No |  |
| 38 | If yes, from whom you have faced any kind of abuse? **(Multiple response)**  What are they? | 1. Husband 2. In-laws 3. Husband’s family 4. Society 5. Relatives 6. Others   ……………………………. |  |
| 39 | What are your suggestions/ recommendations to ease the problems faced during infertility phase? | Answer………. |  |

| **ANNEX 2- REPORTED BY THE RESPONDENTS AND VERIFIED BY THE RESEARCHER** | | |
| --- | --- | --- |
| **Q.N.** | **Questions** | **Response** |
| **Treatment related questionnaires** | | |
| 1 | Type of infertility | 1. Primary 2. Secondary |
| 2 | Duration of infertility | 1. Less than 2 years 2. Between 2-5 years 3. More than 5 years |
| 3 | Factor of infertility | 1. Male factor 2. Female factor |
| 4 | Treatment procedure | 1. TI/OI 2. IUI (Husband sperm) 3. IUI (Donar sperm) 4. IVF (by couple) 5. IVF (Donor sperm) 6. IVF (Egg donation) 7. ICSI with husband sperm 8. ICSI with donor sperm |
| 5 | Has the patient been under any treatment before? If yes, how many times? | 1. Yes 2. No   If yes ……. Times  What are they?........................... |
| 6 | Duration of treatment | 1. Less than 2 years 2. Between 2-5 years 3. More than 5 years |

| **ANNEX 3- STANDARD TOOL (DASS SCALE)** | | | | | | |
| --- | --- | --- | --- | --- | --- | --- |
| **Q. N** | **QUESTIONS** | **Never (0)** | **Sometimes (1)** | **Often (2)** | **Almost always (3)** | **Scoring**  **criteria** |
| 1 | I found it hard to wind down |  |  |  |  | S |
| 2 | I was aware of dryness of my mouth |  |  |  |  | A |
| 3 | I couldn't seem to experience any positive feeling at all |  |  |  |  | D |
| 4 | I experienced breathing difficulty (e.g., excessively rapid breathing, breathlessness in the absence of physical exertion) |  |  |  |  | A |
| 5 | I found it difficult to work up the initiative to do things |  |  |  |  | D |
| 6 | I tended to over-react to situations |  |  |  |  | S |
| 7 | I experienced trembling (e.g., in the hands) |  |  |  |  | A |
| 8 | I felt that I was using a lot of nervous energy |  |  |  |  | S |
| 9 | I was worried about situations in which I might panic and make a fool of myself |  |  |  |  | A |
| 10 | I felt that I had nothing to look forward to |  |  |  |  | D |
| 11 | I found myself getting agitated |  |  |  |  | S |
| 12 | I found it difficult to relax |  |  |  |  | S |
| 13 | I felt down-hearted and blue |  |  |  |  | D |
| 14 | I was intolerant of anything that kept me from getting on with what I was doing |  |  |  |  | S |
| 15 | I felt I was close to panic |  |  |  |  | A |
| 16 | I was unable to become enthusiastic about anything |  |  |  |  | D |
| 17 | I felt I wasn't worth much as a person |  |  |  |  | D |
| 18 | I felt that I was rather touchy |  |  |  |  | S |
| 19 | I was aware of the action of my heart in the absence of physical exertion (e.g., sense of heart rate increase, heart missing a beat) |  |  |  |  | A |
| 20 | I felt scared without any good reason |  |  |  |  | A |
| 21 | I felt that life was meaningless |  |  |  |  | D |
